# Study of waterpipe smoking topography in Fars province of Iran

**DOI:** 10.1038/s41598-024-54973-2

**Published:** 2024-02-23

**Authors:** S. Hosseini, G. Karimi

**Affiliations:** 1 Department of Chemical Engineering, Shiraz University, Shiraz, 7134851154 Iran; 2 Department of Chemical Engineering, University of Larestan, Larestan, Iran

**Keywords:** Waterpipe smoking, Hookah, Narghile, Topography, Tobacco, Waveform, Public health, Psychology and behaviour

## Abstract

Despite a sharp increase in the use of the waterpipe (WP) has been noted recently in Iran, no information is available for the smoking behavior and topography parameters. The present study is intended to obtain the inhalation and smoking topography parameters for the Iranian WP smokers. The smoking data collected from 122 smoking sessions, including 192 WP smokers in the Iranian Fars province have been used to perform smoking topography assessments. The influence of demographic and smoking parameters on puffing data is obtained. Results have indicated that gender and tobacco type strongly affect puff volume and duration. Women smokers inhale smaller volume of smoke than men and puff duration is significantly increased for regular smokers than occasional smokers. However, the results of the present study have not revealed a major effect of age, residence and setting on the puffing behavior.

## Introduction

Waterpipe (WP) smoking has been a very old traditional habit in Middle Eastern and South Asian countries including Iran^1^. In the early 1990’s, however, this method of tobacco smoking has increased in popularity and spread into other parts of the world, including the U.S., Europe and some South American countries^2^. The WP, also known internationally as Hookah, Narghile, Hubble-Bubble or Shisha, is usually referred to as Ghalyan in Iran^3^.

A typical WP consisted of a head (with holes in the bottom), a body, a water bowl and a flexible hose with a mouthpiece. The usual course of smoke in a WP is through a tube traveling from the top of the WP (head) where the burning tobacco is located to the body, descending into the water bowl where it bubbles through, and eventually through the flexible tube and mouthpiece where the smoke inhalation occurs. WP smoking is typically performed in groups, with the same mouthpiece passed from person to person.

Although the WP body, water bowl and hose are manufactured in a variety of sizes, shapes and style, there are two common head types depending on the tobacco used. When Moassel head is used, smokers fill the head with a fairly deep tobacco mixture (approximately 3 cm in height, 10–15 g weigt), and cover it with a perforated aluminum foil for air passage. The already burning charcoal is placed on the top of the aluminum foil to initiate the smoke. With Ajami head, on the other hand, the pre-shredded and dried Ajami tobacco is mixed with a small amount of water to make a moldable matrix which is then shaped into a small mound atop a shallow head. The burning charcoal is placed directly on the top of the moisturized tobacco such that both tobacco and charcoal are exposed directlyto the surrounding air to sustain the smoke generation^4^.

Many researchers have devoted their effort on studying WP smoking due to its rising popularity and the associated adverse health effect^5–7^. Machine smoking protocols have been developed based on smoking behavior and various constituents of WP smoke (e.g. carbon monoxide (CO), polycyclic aromatic hydrocarbons (PAHs), aldehydes, etc.) were identified^8–11^. Rakower and Fatal^12^ were the first to develop and use a smoking machine to assess the mainstream smoke (MSS) in a WP. Several modern WP smoking machine studies were conducted in Lebanon^8,13,14^, Germany^15–18^ and Switzerland^19,20^.

The smoking machine studies have employed different smoking topography parameters such as puff frequency, puff volume, inter-puff interval (IPI) and session duration^13^. As a result, the results obtained and conclusions made are not in general agreement. For instance, the average puff volume has been changed from 300 ml^4^ to 530^8^ and to 1020 ml^21^ or puff duration has been changed from 3s^4^ to 2.6s^8^ and to 3.9s^21^. Also, the IPI has been varied from 30s^4^ to 17s^8^ and to 15.3s^21^. Such variations can be attributed to differences in WP design, instrumentation, data analysis, smoker characteristics^22^ (e.g., gender, age and prior smoking experience) and settling (home, café, public outdoor places, etc.). As a result, the reported amount of toxic components such as tar, nicotine, carbon monoxide and other carcinogens in the smoke are significantly different.

It should be noted that WP and cigarette smoking characteristics are different. The paper cigarettes contain materials that control the burning rate. On the other hand, the manufacturing and packing of the tobacco blends in cigarettes are in such a way that tobacco consumption remains almost uniform during the whole smoking session. Therefore a fixed topographical smoking regime which is standardized by Federal Trade Commission (FTC) is normally adopted to generate the cigarette smoke samples^23^. On the contrary, the topographical parameters during a WP smoking session normally change due to the effect of non-uniform distribution and consumption of tobacco and charcoal. In addition, other smoking parameters such as WP size, shape, style, types of tobacco and charcoal used as well as sharing status can significantly affect the smoking topography. To this end, more comprehensive topographical data are needed for programming laboratory-based WP smoking machines and to perform mathematical modeling of transport phenomena during WP smoking^24^.

The first detailed topographic study was conducted by Shihadeh et al.^25^ using 52 volunteer WP smokers. They reported that the mean number of puff cycles per average session (61 min duration) was 171 with a puff volume of 530 ml, puff duration of 2.6 s and IPI of 17 s. Their study was conducted in a café in the Hamra neighborhood of Beirut, Lebanon. Other smoking settings, place of use (e.g. home and public outdoor places) and smoker characteristics (e.g., gender, age, and prior smoking experience) can affect smoking topography. These settings and variables such as smoking frequency (occasional or regular), sharing status (shared or non-shared), residence (urban or rural), type of tobacco used (Ajamy or Moassel) were not considered in their study. In addition, the smoking sessions were not sampled in their entirety, which would have eliminated the need to extrapolate the puff parameters. Therefore it is essential to perform a more comprehensive investigation on the WP smoking topography.

The main objective of the present study is to assess the influence of demographic parameters (age, gender, residence and setting) and smoking parameters (smoking frequency, type of tobacco used and sharing status) on puffing behavior and pattern of inhalation during WP smoking. Information were collected from volunteers resided in Shiraz (one of the largest cities located in the southern part of Iran) and a couple of other small nearby cities/villages. The information obtained from this study can be used as a guideline to perform WP smoking behavioral studies in other Iranian regions and for design and simulation of WP smoking machines.

## Methods

### Study design and instrumentation

In the present study, WP smoking topography is obtained by visual observations, interviews and data collection during random visits to local cafés and through field surveys. The key parameters considered in the survey are age, gender, smoking frequency, sharing status and the type of tobacco used. To evaluate the effect of these factors, the smoking topography parameters for each smoking session was determined by two methods. In the first method, the parameters (except puff volume) were obtained by visual observation of the smokers and recording the WP sound during the smoking session. The times corresponding to puff duration and IPI for each smoker were recorded by two individual observers using separate stopwatches with an estimated accuracy of 0.2 s per puff. Also, the starting and ending times of the smoking session were recorded. The accuracy and reliability of the collected data were later checked again by listening to the sound recordings. In the second method, a calibrated mass flow meter (Alicat M Standard Series: M-20SLPM-D) was attached to the inlet of the WP hose, far from the mouthpiece to measure the instantaneous volumetric flow rates of smoke. The collected data were stored in the instrument’s memory. Fortunately, there was a very high positive response from individuals approached for this method of assessment and in fact the smokers indicated that they sensed very little difference between smoking with and without the flow meter attached.

All procedures performed in studies involving human participants were in accordance with the ethical standards of the Helsinki Declaration and involved no additional risk to the smokers. Also, in compliance with the approach used by Chapman et al.^26^, no communication was made with the smokers prior the smoking sessions.

To complete the smoking topography, participants were also asked to fill a survey form giving their personal information (e.g. age, gender, favorite tobacco flavor and smoking frequency). The survey was brief, since longer surveys could have disrupted the business and led to poor response from customers who wanted to enjoy their times. The written informed consent was obtained from all the participants. This study protocol was approved by an ethics committee at Shiraz University and carried out in accordance with guidelines of that committee.

### Calculation procedures

In the first measurement method (stopwatches and sound recordings), the average smoking topography including number of puffs, puff duration and IPI were obtained simply by using the recorded data. In the second method, instantaneous volumetric flow rate data were retrieved from the flow meter and used to obtain the smoking topography.

**Figure 1:**
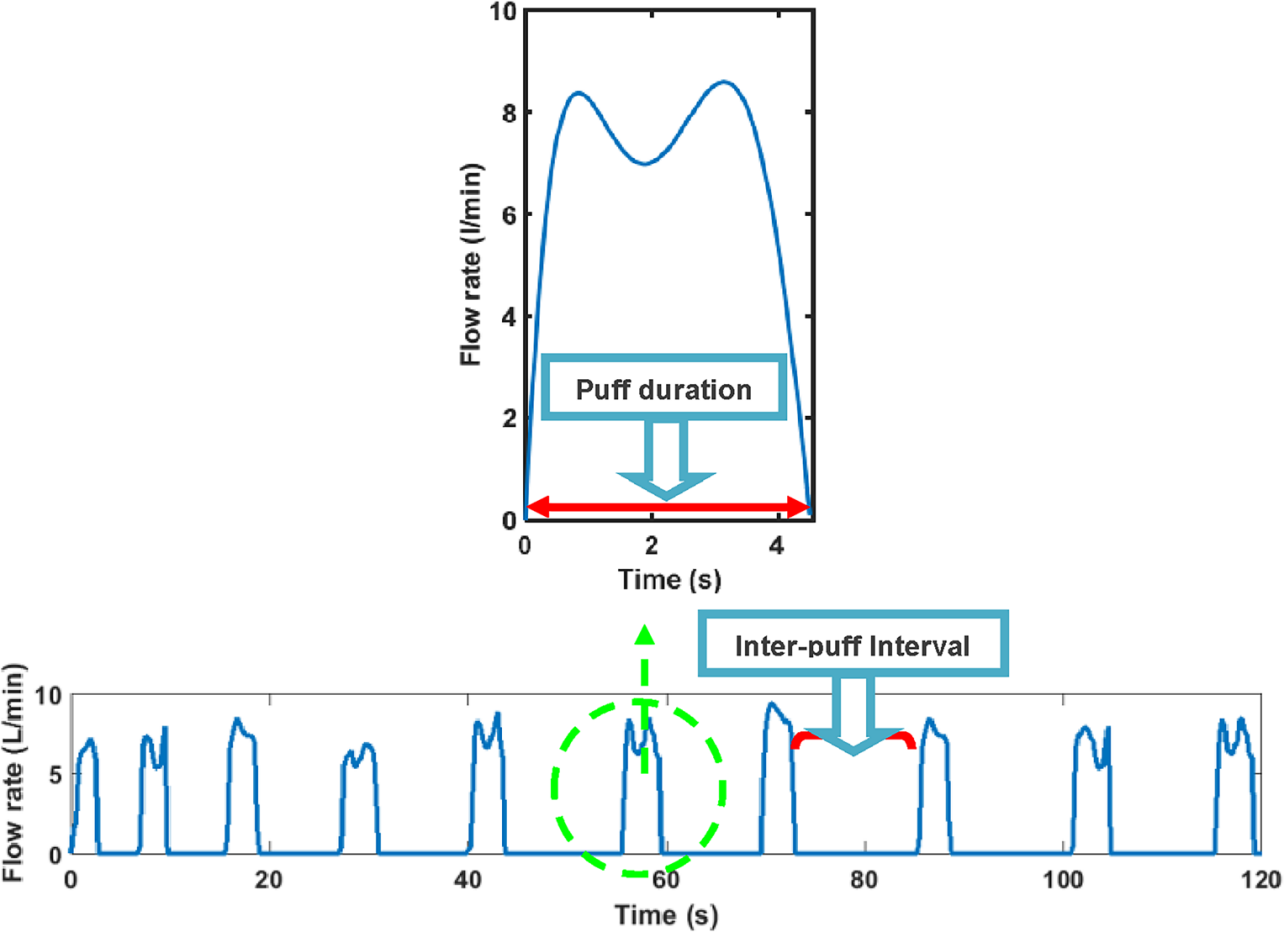
Typical puffing waveform and common smoking topography parameters.

A typical puffing waveform obtained in the second method is shown in Fig. 1. For an arbitrary puff $i$ in this waveform, the inhaled volume, ${V}_{i}$, can be determined by integration of the instantaneous volumetric flow rate, ${q}_{i}\left(t\right)$, over time, $t$ based on Eq. (1).

1 $${V}_{i}={\int }_{{t}_{s}\left(i\right)}^{{t}_{e}\left(i\right)}{q}_{i}\left(t\right)dt$$

where ${t}_{s}(i)$ and ${t}_{e}\left(i\right)$ are the starting and ending times of puff $i$, respectively (e.g. ${t}_{e}(i)-{t}_{s}(i)$ is equal to puff duration). The mean flow rate for puff $i$, ${Q}_{i}$, is then calculated from Eq. (2).

2 $${Q}_{i}=\frac{{V}_{i}}{{t}_{e}(i)-{t}_{s}(i)}$$

Statistical methods can be used to estimate average puff parameters such as puff duration, puff volume, IPI and puff frequency for each smoking session.

It is expected that the smoking behavior (or topography) to change during a smoking session. To address this point, the first 30 min of each recorded data (in the second method) was divided into 4 identical time intervals (7.5 min each) and the average smoking topography for each time interval was obtained and examined.

### Data analysis

Independent sample t-test was used to examine the differences in smoking topography parameters between demographic characteristics and smoking parameters such as gender, smoking frequency, WP sharing status, residence (urban or rural), tobacco type and the method of measurement. Also, one-way analysis of variance (ANOVA) was used to examine the difference in smoking topography parameters among demographic characteristics and smoking parameters such as age (e.g. < 25, 25–40, > 40 years) and setting (home, café, dormitory and outdoor). In addition, the effect of WP sharing status on the puff behavior during smoking sessions was analyzed by using univariate within-subject ANOVA. Differences between the mean values were examined using Tukey’s Honestly Significant Difference^27^ and considered significant if P-value was less than 0.05 (or 95% confidence interval). We use the Kolmogorov–Smirnov (KS) test to show that the Normal distribution is a good fit to each group of data sets. The p-values of KS test for the each group are great than Significance Level (α = 0.05). Therefore, the Normal distribution is a suitable model for each group of data. The collected data were analyzed using IBM SPSS Statistics version 21.

### Participation and setting

Data collection took place between November 2015 and January 2020 in the Iranian southern province of Fars. Participants were from Shiraz (the center of province), four nearby cities (Marvdasht, Zarqan, Lamerd and Lar) and four nearby villages (Kuh Sabz, Badaki, Deh Sheykh and Kowreh). The participants were interviewed and their personal information and smoking data were collected at their homes, cafés, student dormitories or outdoors (tourism attractions such as Persepolis and Shiraz Qur'an Gate). The three Shiraz cafés were selected from different neighborhoods and the single café is chosen from the city of Marvdasht. Also, smoking information was obtained from both male and female students resided in Shiraz University dormitories. Most data collection occurred during weekend nights (particularly in cafés and tourism attractions) because of the large number of WP smokers were available during those periods.

## Results

### Demographic characteristics

Overall, 192 WP smokers were participated in the study. Some of the smoking sessions were excluded because the smokers were unable to finish the smoking session for any particular reason. In total, the data from 122 smoking sessions were compiled. Of 122 sessions examined, 77 sessions were non-shared (single smoker) and 45 sessions were shared (multi-users). Details of demographic characteristics and smoking information of the participants are given in Tables 1 and 2.

**Table 1:** Demographic characteristics and smoking information of the participants (non-shared smoking).

Characteristics	Number of participants	Percentage
Age		
< 25	29	37.7
25–40	32	41.5
> 40	16	20.8
Gender		
Female	36	46.8
Male	41	53.2
Frequency of smoking		
Occasional	31	40.3
Regular (weekly)	46	59.7

**Table 2:** Demographic characteristics and smoking information of the participants.

Characteristics	Number of participants (percentage)		Percentage
	Non-shared	Shared	
Residence			
Urban	63 (51.6)	36 (29.5)	81.1
Rural	14 (11.5)	9 (7.4)	18.9
Setting			
Home	12 (9.8)	8 (6.6)	16.4
Café	36 (29.5)	15 (12.3)	41.8
Dormitory	12 (9.8)	9 (7.4)	17.2
Outdoor	17 (13.9)	13 (10.7)	24.6
Type of tobacco used			
Ajamy	29 (23.8)	17 (13.9)	37.7
Moassel	48 (39.3)	28 (23.0)	62.3
Method			
Stopwatch	35 (28.7)	19 (15.6)	44.3
Flow meter	42 (34.4)	26 (21.3)	55.7

### The effect of various factors on topographical parameters

#### Non-shared WP smoking

**Figure 2:**
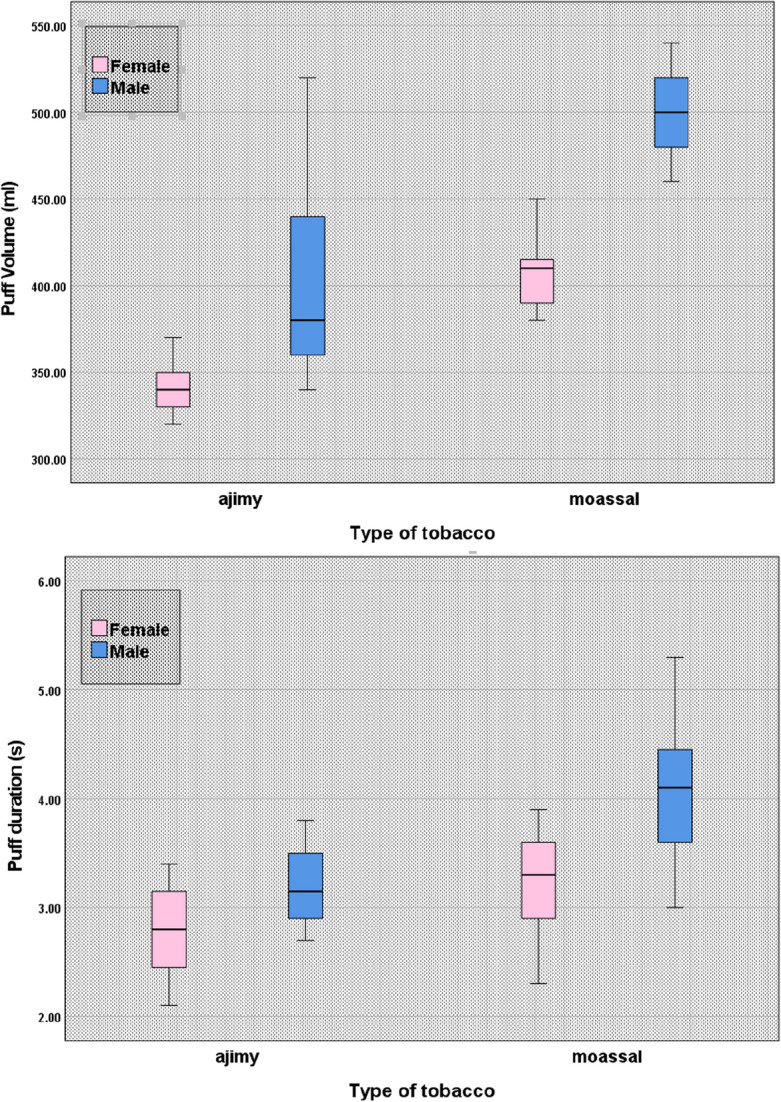
Effect of smoker gender and type of tobacco on puff volume and duration.

In the present study, the smoking data across the entire sessions were analyzed and the influences of participants’ characteristics and smoking parameters on smoking topography are obtained. As indicated in Table 3, there is a significant difference in puff volume between female and male participants (381.90 ± 43.24 ml versus 464.50 ± 83.06 ml; P < 0.005) and puff duration (3.07 ± 0.49 s versus 3.77 ± 0.67 s; P < 0.0001) (mean ± SD). This means that the average puff duration is 22.80% longer for male than female and as a result, on average, larger amounts of smokes being inhaled by male participants (21.63% larger). Measurements also show that the participants’ habits for WP smoking considerably affects the puff duration (P < 0.0001).Information obtained in this study has revealed that puff duration is significantly longer for regular smokers than occasional smokers (mean ± SD: 3.77 ± 0.58 s versus 2.95 ± 0.52 s, or 27.8% longer). In addition, the type of tobacco used by the participants has considerable influence on the puff volume and duration (P < 0.0001). For instance the collected data show that the puff volume and duration (mean ± SD) are statistically significantly larger for Moassal than Ajamy (486.6 ± 70.19 ml versus 390.00 ± 60.50 ml and 3.70 ± 0.68 s versus 3.01 ± 0.45 s, respectively). Overall, the effect of gender and type of tobacco on puff volume and puff duration is remarkable as can be seen in Fig. 2.

**Table 3:** The influence of participants’ characteristics and smoking parameters on smoking topography (mean (SD)) of non-shared WP.

Characteristics	Category			P-value	
Age^‡^	< 25	25–40	> 40		
Number of puffs	107.27 (20.24)	122.28 (30.84)	106.56 (25.14)	0.061	
Puff volume (ml)	421.20 (64.99)	450.00 (97.67)	426.0 (61.50)	0.565	
Puff duration (s)	3.41 (0.66)	3.49 (0.68)	3.39 (0.79)	0.864	
IPI (s)	13.80 (2.18)	14.20 (1.97)	14.93 (2.42)	0.247	

Characteristics	Category		P-value		
Gender^†^	Female	Male			
Number of puffs	107.11 (25.24)	118.85 (27.32)	0.055		
Puff volume (ml)	381.90 (43.24)	464.50 (83.06)	0.001		
Puff duration (s)	3.07 (0.49)	3.77 (0.67)	0.000		
IPI (s)	14.33 (2.16)	14.09 (2.18)	0.630		

Characteristics	Category		P-value		
Frequency^†^	Occasional	Regular (weekly)			
Number of puffs	110 .39 (21.07)	115.37 (30.19)	0.428		
Puff volume (ml)	435.30 (92.34)	421.10 (67.32)	0.598		
Puff duration (s)	2.95 (0.52)	3.77 (0.58)	0.000		
IPI (s)	14.58 (2.34)	13.94 (2.02)	0.211		

Characteristics	Category		P-value		
Residence^†^	Urban	Rural			
Number of puffs	113.03 (27.61)	114.85 (24.01)	0.993		
Puff volume (ml)	427.80 (79.25)	464.00 (41.59)	0.575		
Puff duration (s)	3.40 (0.64)	3.762 (0.86)	0.175		
IPI (s)	13.92 (2.15)	15.45 (1.78)	0.561		

Characteristics	Category				P-value
Setting^‡^	Home	Café	Dormitory	Outdoor	
Number of puffs	115.17 (17.99)	112.64 (24.56)	112.92 (32.98)	113.94 (33.63)	0.947
Puff volume (ml)	456.00 (52.25)	425.80 (62.41)	420.00 (120.31)	480.00 (45.83)	0.167
Puff duration (s)	3.66 (0.75)	3.35 (0.64)	3.18 (0.57)	3.65 (0.76)	0.154
IPI (s)	14.52 (2.58)	14.36 (2.07)	13.40 (1.82)	14.20 (2.31)	0.434

Characteristics	Category		P-value		
Type of tobacco^†^	Ajamy	Moassel			
Number of puffs	116.72 (26.6)	111.3 (27.07)	0.397		
Puff volume (ml)	390.00 (60.50)	486.60 (70.19)	0.000		
Puff duration (s)	3.01 (0.45)	3.70 (0.68)	0.000		
IPI (s)	14.51 (2.10)	14.01 (2.20)	0.326		

^†^Assessed by independent sample t-test.

^‡^Assessed by one-way analysis of variance (ANOVA).

It is noteworthy that the results did not reveal significant effect of age, residence, setting and sampling methods on the puffing behavior (P > 0.05 for all).

#### Shared WP smoking

Table 4 indicates the influence of smokers’ characteristics and smoking parameters on the smoking topography during shared smoking. Results show that puff volume is statistically significantly larger for Maossal tobacco than that of Ajamy tobacco (554.2 ± 61.49 ml versus 421.40 ± 38.05 ml) and similarly, puff duration is longer for Moassal tobacco than that of Ajamy tobacco (4.13 ± 0.41 versus 3.58 ± 0.40). However, the results have shown that the insignificant effects of residence and setting on puffing behavior (P > 0.05 for all).

**Table 4:** The influence of participants’ characteristics and smoking parameters on smoking topography (mean (SD)) of shared WP.

Characteristics	Category		P-value		
Residence^†^	Urban	Rural			
Number of puffs	128.17 (24.92)	137.47 (24.01)	0.361		
Puff volume (ml)	523.2 (86.43)	492.50 (47.87)	0.501		
Puff duration (s)	3.95 (0.50)	3.83 (0.46)	0.537		
IPI (s)	7.54 (1.71)	9.36 (2.67)	0.055		

Characteristics	Category				P-value
Setting^‡^	Home	Café	Dormitory	Outdoor	
Number of puffs	133.91 (24.21)	131.90 (30.23)	130.02 (24.99)	125.49 (28.31)	0.902
Puff volume (ml)	554.00 (96.59)	502.20 (72.59)	546.00 (113.49)	494.30 (56.82)	0.503
Puff duration (s)	4.12 (0.74)	3.90 (0.43)	3.90 (0.40)	3.92 (0.49)	0.640
IPI (s)	9.11 (2.15)	7.50 (1.89)	8.01 (1.50)	7.55 (2.37)	0.292

Characteristics	Category		P-value		
Type of tobacco^†^	Ajamy	Moassel			
Number of puffs	129.82 (23.68)	130.16 (29.17)	0.968		
Puff volume (ml)	421.40 (38.05)	554.2 (61.49)	0.000		
Puff duration (s)	3.58 (0.40)	4.13 (0.41)	0.000		
IPI (s)	7.99 (2.29)	7.85 (1.92)	0.826		

^†^Assessed by independent sample t-test.

^‡^Assessed by one-way analysis of variance (ANOVA).

### Changes in puffing behavior during the smoking session

**Figure 3:**
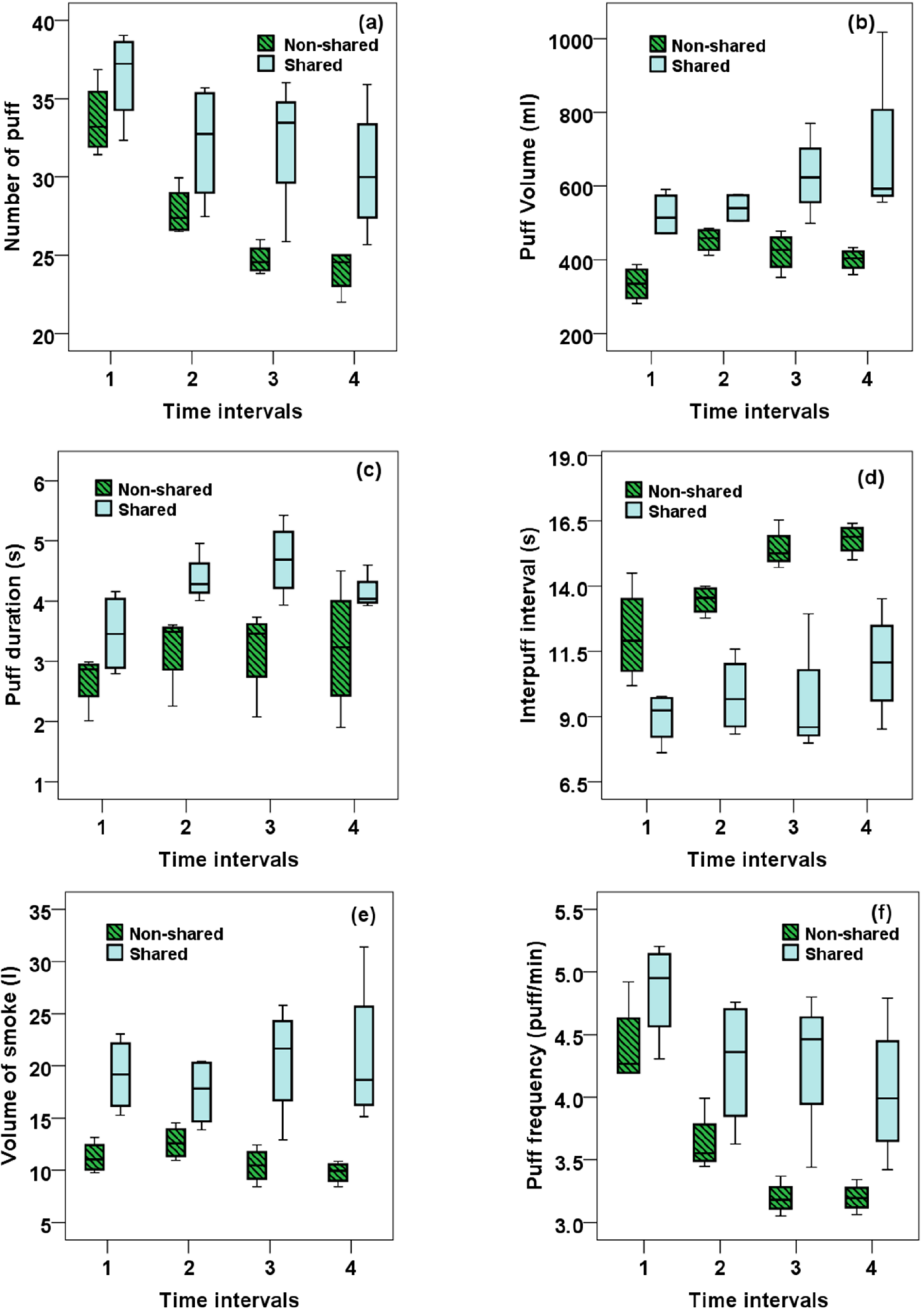
Average smoking topography during the first 30 min of smoking for non-shared and shared smoking.

As mentioned in section "Calculation procedures", to obtain time varying smoking behavior, the first 30 min of each session was divided into 4 identical time intervals (7.5 min each) and the average smoking topography for each time interval was obtained. Figure 3a–f shows average smoking topography at different time intervals in the first 30 min of smoking session for non-shared and shared smoking.

Statistical examinations of the collected data shown in Fig. 3 indicate that the smoking topography undergoes significant changes (in term of the number of puffs, IPI and puff frequency) during the course of one smoking session for both shared and non-shared smokers. However, the changes in the puff volume, puff duration and mean volume of smoke in each time interval are shown to be statistically insignificant (P > 0.05).

As seen from Fig. 3a, there is a significant drop in the mean number of puffs as WP smoking proceeds (P < 0.0001). In fact, for non-shared and shared smoking, the mean number of puffs is declined by 28.66% and 16.64% during the first 30 min of smoking, respectively. Results also indicate that IPI increases considerably during the course of one smoking session (P < 0.005). As seen from Fig. 3d, for non-shared and shared smoking, the mean IPIs are increased by 30.28% and 23.14% during the first 30 min of smoking, respectively. As a result, Fig. 3f shows that for non-shared and shared smoking, the mean puff frequency is decreased by 27.53% and 16.63% during the first 30 min of smoking, respectively.

The collected data from 122 smoking sessions have indicated that on average a smoking session takes 34 ± 3 min for non-shared and 41 ± 4 min for shared smoking.

The summary of smoking topography parameters are listed in Table 5 for both non-shared and shared smoking session.

**Table 5:** Smoking topographical parameters during various time intervals.

Parameters	Interval 1	Interval 2	Interval 3	Interval 4
Non-shared				
Puff duration	2.68	3.21	3.18	3.22
IPI	12.12	13.46	15.44	15.08
Puff frequency	4.41	3.63	3.19	3.20
Puff volume	355.79	461.64	455.06	441.22
Shared				
Puff duration	3.46	4.38	4.68	4.15
IPI	8.96	9.81	9.53	11.04
Puff frequency	4.85	4.28	4.29	4.05
Puff volume	501.70	532.69	594.88	649.69

### Inhalation pattern

A typical smoking waveform for an individual smoker was shown in Fig. 1. It is expected that the smoking waveform vary during a smoking session as well as from one smoker to another. Even two consecutive smoking waveforms from an individual are expected to be different. Therefore, one needs to combine all smoking characteristics from all the smokers together to obtain a generalized smoking waveform.

In the present study the collected waveform data from all smoking sessions for each of the time intervals are combined and the mean smoking waveforms are obtained.

**Figure 4:**
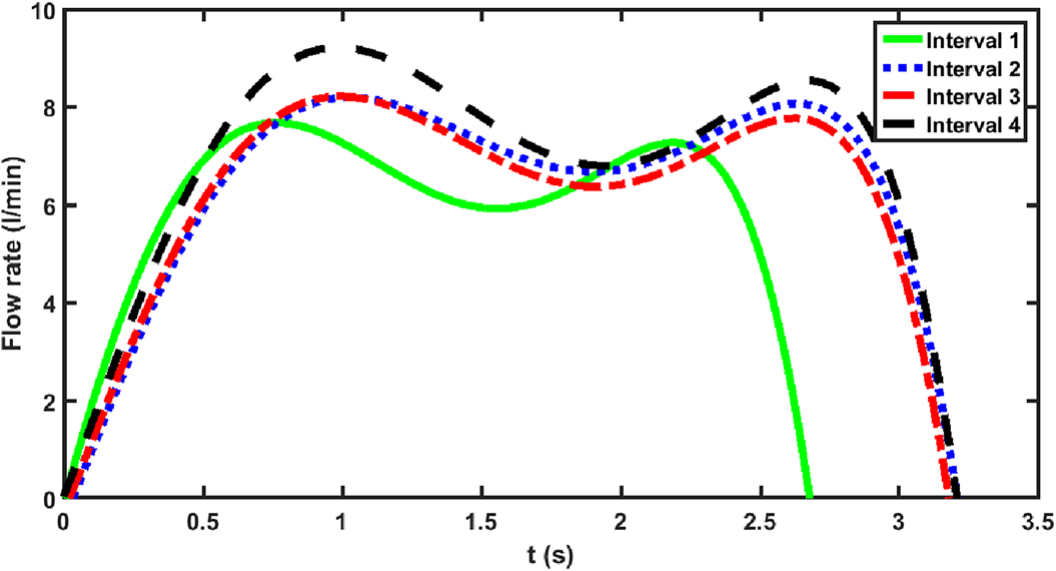
The overall mean smoking waveforms for different time intervals for non-shared smoking.

**Figure 5:**
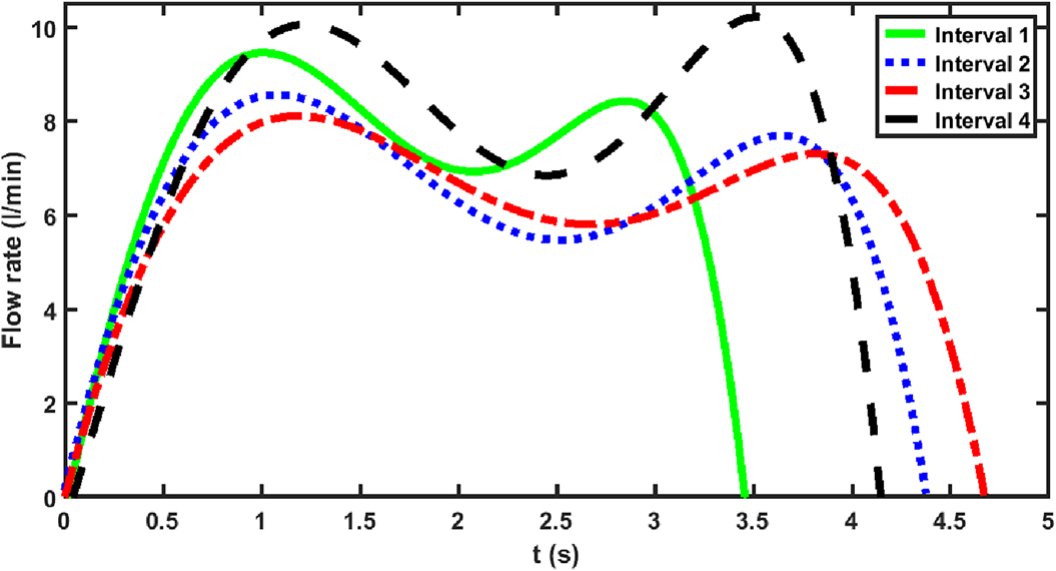
The overall mean smoking waveforms for different time intervals for shared smoking.

Figures 4 and 5 show the generalized mean smoking waveforms at different time intervals for non-shared and shared smoking, respectively. It is evident from these plots that the generalized mean smoking behavior changes not only from one time interval to another but also changes depending on the sharing status.

## Discussion

The influence of demographic parameters and smoking parameters on puffing data was assessed. Statistical results show that there are significant differences in puff volume and duration between female and male smokers. Similar to our results, other studies also observed that the men have a greater puff volume and puff duration than women^28–30^. Also, regular smokers have a significantly larger puff duration than occasional smokers^30^. On the other hand, results did not reveal a significant effect of age, residence, setting and sampling methods on puffing behavior. Surprisingly, all parameters did not have a statistically significant effect on IPI and number of puff which is somewhat different from the results of other studies. One limitation of these studies is that their laboratory-scale conduction may create undesirable influence on the user puffing behavior. It should be noted that few studies, with inconsistent results, have investigated WP smoking topography.

In this study, the changes in puffing behavior and pattern of inhalation during the WP smoking session were also investigated. The average patterns of inhalation and topographical parameters at different time intervals of a smoking session were determined for non-shared and shared smoking. It can be concluded that within the range of conditions studied, the mean number of puffs and puff frequency are declined and IPI is increased during the smoking sessions. Indeed, the tobacco temperature in the short IPIs is higher^4^ because there is less time for the tobacco to cool between puffs. As a result, smoke toxic content increases by increasing the tobacco temperature. The changes in puff volume, puff duration and volume of smoke are shown to be insignificant during the smoking sessions. Some of findings reported in this study are consistent with those of previous published studies. Nevertheless, the findings of this study should be considered in light of several limitations. One such limitation is the small number of subjects participating in the study. Another limitation is that subjects were selected from the southern province of Fars (Iran). Ultimately, it must be noted that more work is needed to understand the WP smoking behavior and as a result its health risks.

## Conclusion

The present study was conducted to develop a preliminary model of inhalation and topographical parameters for use in laboratory smoking machine studies. Statistical results show that the volumes of smoke of females is smaller than male smokers and as a result, the puff volume and duration are smaller. Puff duration and volume are statistically significantly larger for Moassal tobacco than those of Ajamy tobacco. This study is the first to document the puffing behavior during WP smoking.

## Data Availability

All data generated or analyzed during this study are available on reasonable request from the corresponding author.

## List of symbols

$$i$$: Puff number
${q}_{i}\left(t\right)$: Instantaneous volumetric flow rate for puff $i$, ml/s
${Q}_{i}$: Mean flow rate for puff $i$, ml/s
$t$: Time of inhalation, s
${t}_{e}(i)$: Ending time of puff $i$, s
${t}_{s}(i)$: Starting time of puff $i$, s
${V}_{i}$: Inhaled volume of puff $i$, ml
